# 568 Virtualization of In-school, Teacher-Directed Prevention Programs for Children

**DOI:** 10.1093/jbcr/irac012.196

**Published:** 2022-03-23

**Authors:** Jessica L Banks

**Affiliations:** Jessica Banks, Easton, Pennsylvania

## Abstract

**Introduction:**

While remote/virtual teaching has been used for many years, Covid-19 increased the need for quality educational programs in virtual format. BPN has two in-school, school-based programs, *Flicks Fire and Burn Safety* for grades 1-5 and *The Great Escape* for grades 6-8. These teacher-directed programs cover essential topics such as home fire safety, stop drop and roll, youth fire misuse, and home fire escape. They have, historically, been deployed as hard copy curriculum, student workbooks, and associated educational videos, which were physically mailed directly to classrooms within the BPN’s 24-county service area. When Covid-19 became the new reality, the need to pivot to a different type of program delivery became apparent.

**Methods:**

BPN recruited professional educators from targeted grade levels, representing varying geographic locations and socioeconomic backgrounds to recreate these programs in a format that will work in a remote learning environment. This team researched various e-learning platforms to determine what would work best for our content and is commonly used across all school districts. We determined that Google Classroom combined with Bitmoji would best serve our needs. We recreated a set of age-appropriate lessons for each grade level from 1-8. Each grade has information that ties each lesson to the appropriate academic standard for education. We employed multiple content formats based on best practices, including videos, audio tracks, text-based articles, and more. Lastly, we virtualized activities by turning them into on-screen interactive games, puzzles, quizzes, worksheets, etc.

**Results:**

Our result is a virtual program for grades 1-8 conducive to both classroom and remote learning environments. It is linked directly to academic standards for education for each grade level and includes engaging, interactive content in various forms. This format not only gives us the ability to update and add new content quickly it also gives us the ability to expand geographic distribution seamlessly.

**Conclusions:**

By utilizing platforms preferred by professional educators and integrating fire safety and burn prevention lessons into existing mandated educational standards, we can more effectively and cost-efficiently expand quality educational programming to children ages 6 – 14.

